# Supplementary material for: Matrix and analysis metadata standards (MAMS) to facilitate harmonization and reproducibility of single-cell data
Source: bioRxiv. 2023 Mar 7:2023.03.06.531314. Preprint. [Version 1] doi: 10.1101/2023.03.06.531314 (PMC10028847; doi:10.1101/2023.03.06.531314)
Supplement: Supplement 1 [file NIHPP2023.03.06.531314v1-supplement-1.pdf]

bioRxiv preprint doi: https://doi.org/10.1101/2023.03.06.531314; this version posted March 7, 2023. The copyright holder for this preprint (which was not certified by peer review) is the author/funder, who has granted bioRxiv a license to display the preprint in perpetuity. It is made available under aCC-BY 4.0 International license.

SUPPLEMENTARY FIGURES

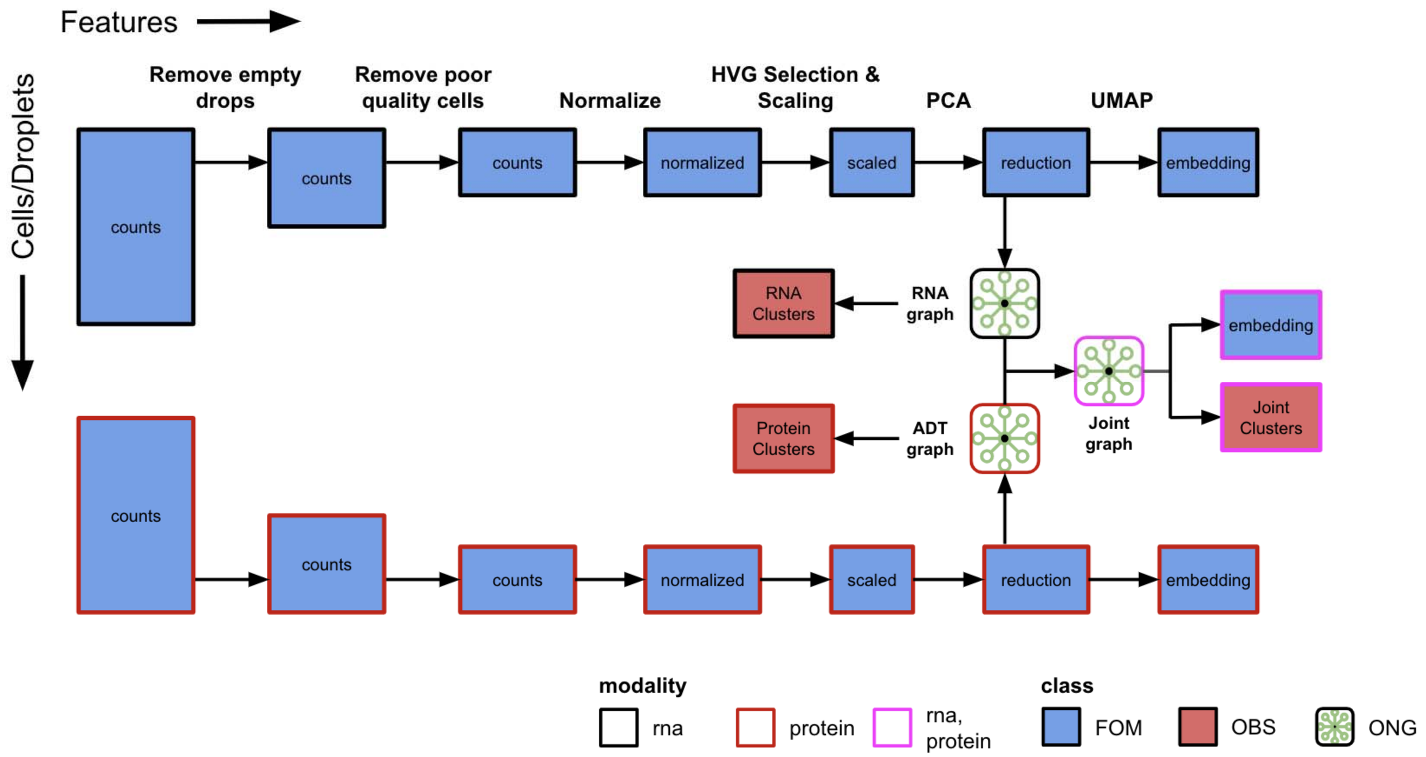

Supplementary Figure 1. Multi-modal single cell RNA-seq and CITE-seq analysis workflow. Several technologies can produce multi-modal data by capturing different biological analytes on the same sets of observations. This workflow demonstrates FOMs and annotations for single cells that are produced by joint profiling of RNA and protein expression using CITE-seq. A separate data matrix is generated for each modality (box color) and will undergo filtering for empty drops and poor-quality cells. Although the same set of cells are retained for analysis in this illustration, different sets of filtering criteria may be applied to each modality independently. Log normalization, scaling, dimensionality reduction, 2-D embedding, and clustering can be performed in parallel for each modality. Further analysis can be performed using a nearest neighbor graph generated by combining the individual neighbor graphs from each modality. A new 2-D embedding and set of cluster labels can be generated with both underlying modalities. The “modality” field can be a list to denote the combination of analytes that were used in the generation of the FOM. Feature annotation and ID classes are not shown.

bioRxiv preprint doi: https://doi.org/10.1101/2023.03.06.531314; this version posted March 7, 2023. The copyright holder for this preprint (which was not certified by peer review) is the author/funder, who has granted bioRxiv a license to display the preprint in perpetuity. It is made available under aCC-BY 4.0 International license.

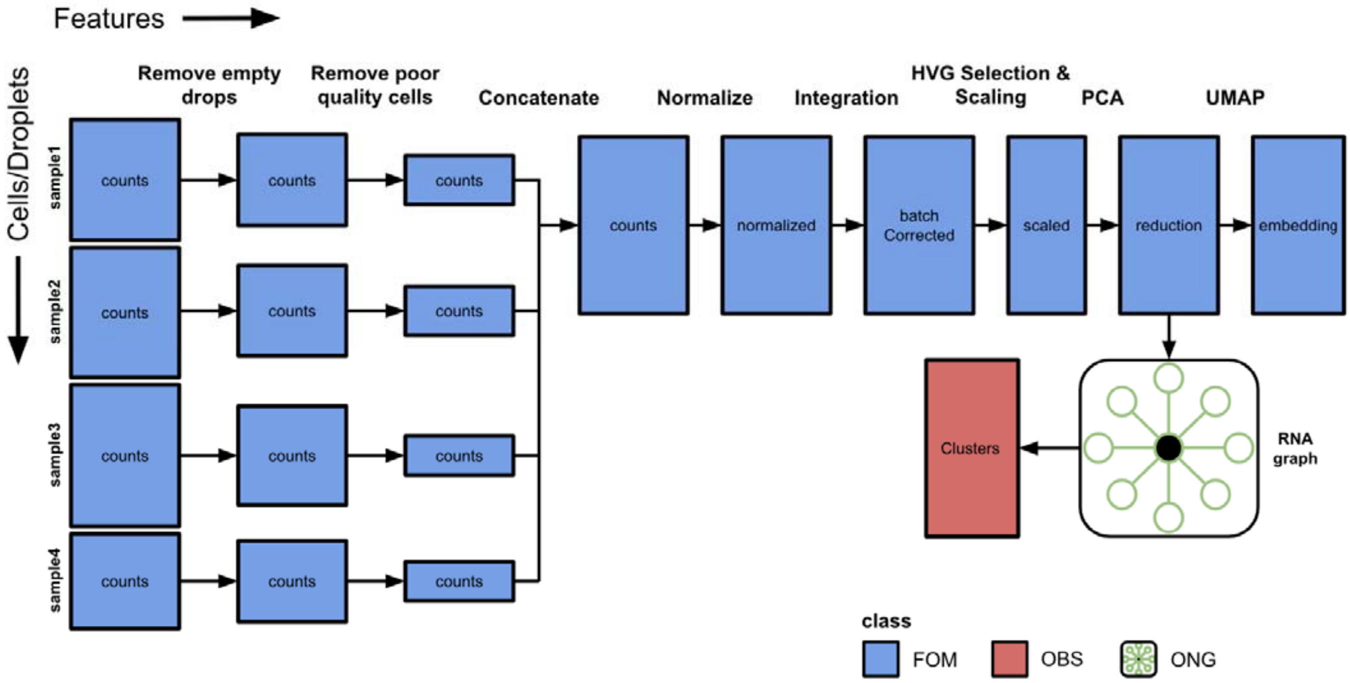

Supplementary Figure 2. Multi-sample single-cell analysis workflow with batch correction. In many datasets, data will be generated for multiple samples and jointly analyzed. This workflow demonstrates data matrices from four samples with different numbers of observations. Empty droplets are identified and removed from each sample individually followed by removal of poor quality cells or artifacts such as doublets/multiplets. Filtered matrices from all samples are then concatenated to produce a combined cell matrix. Note that the order of filtering may be different in various workflows (e.g. filtering of poor quality cells may occur after concatenation). The combined matrix of all samples can be analyzed using standard workflows for normalization, scaling, dimensionality reduction, 2-D embedding, and clustering. In some circumstances, technical differences between samples could produce unwanted clustering. Integration and batch correction methods can be applied at different steps depending on the algorithm or statistical method. In this example, a batch correction algorithm is applied to the log normalized data to produce a new matrix which can be used in subsequent steps. Other variations of this workflow may include using a batch correction or integration method that works on the reduced dimensional matrices to produce a new reduced dimensional object which can subsequently be used to generate a graph.

bioRxiv preprint doi: https://doi.org/10.1101/2023.03.06.531314; this version posted March 7, 2023. The copyright holder for this preprint (which was not certified by peer review) is the author/funder, who has granted bioRxiv a license to display the preprint in perpetuity. It is made available under aCC-BY 4.0 International license.

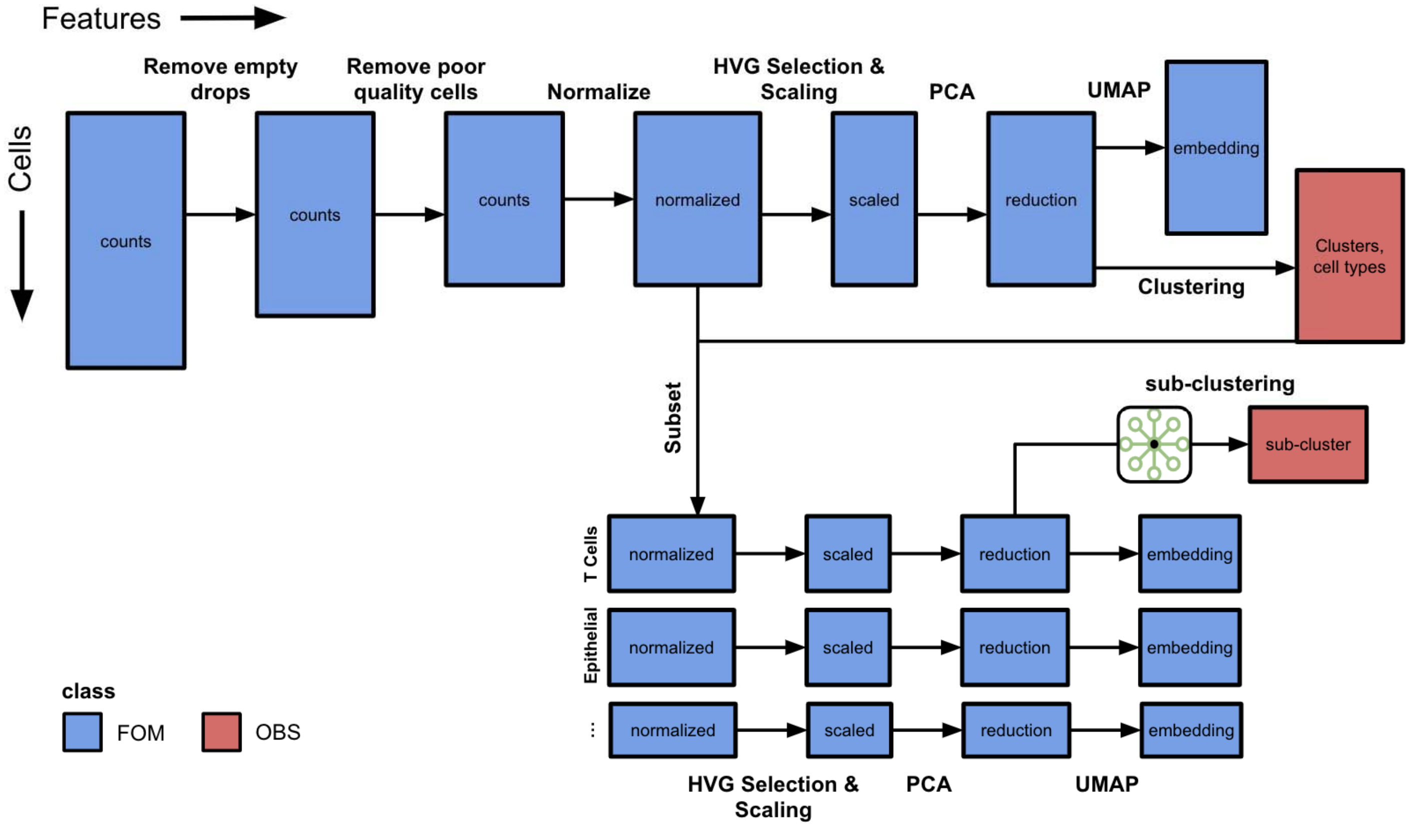

Supplementary Figure 3. Single-cell RNA-seq workflow with additional analysis of biological subsets. During the analysis of real-world datasets, subsets of data are obtained for additional downstream analysis. In this example, a matrix of scRNA-seq data is taken through the standard analysis workflow including filtering, normalization, scaling, dimensionality reduction, and clustering. While this workflow may identify the major cell types present in the dataset, further clustering of individual cell types may reveal additional heterogeneity. The normalized matrix is subsetted using cell type labels derived from clustering. The *obs_subset* field can be used to denote biological subsets of observations such as T-cell, epithelial cell, etc. Each subsetted normalized matrix will undergo a similar workflow of scaling, dimensionality reduction, 2-D embedding, and clustering to identify subpopulations within each major cell type. Feature annotation and ID classes are not shown.

bioRxiv preprint doi: https://doi.org/10.1101/2023.03.06.531314; this version posted March 7, 2023. The copyright holder for this preprint (which was not certified by peer review) is the author/funder, who has granted bioRxiv a license to display the preprint in perpetuity. It is made available under aCC-BY 4.0 International license.

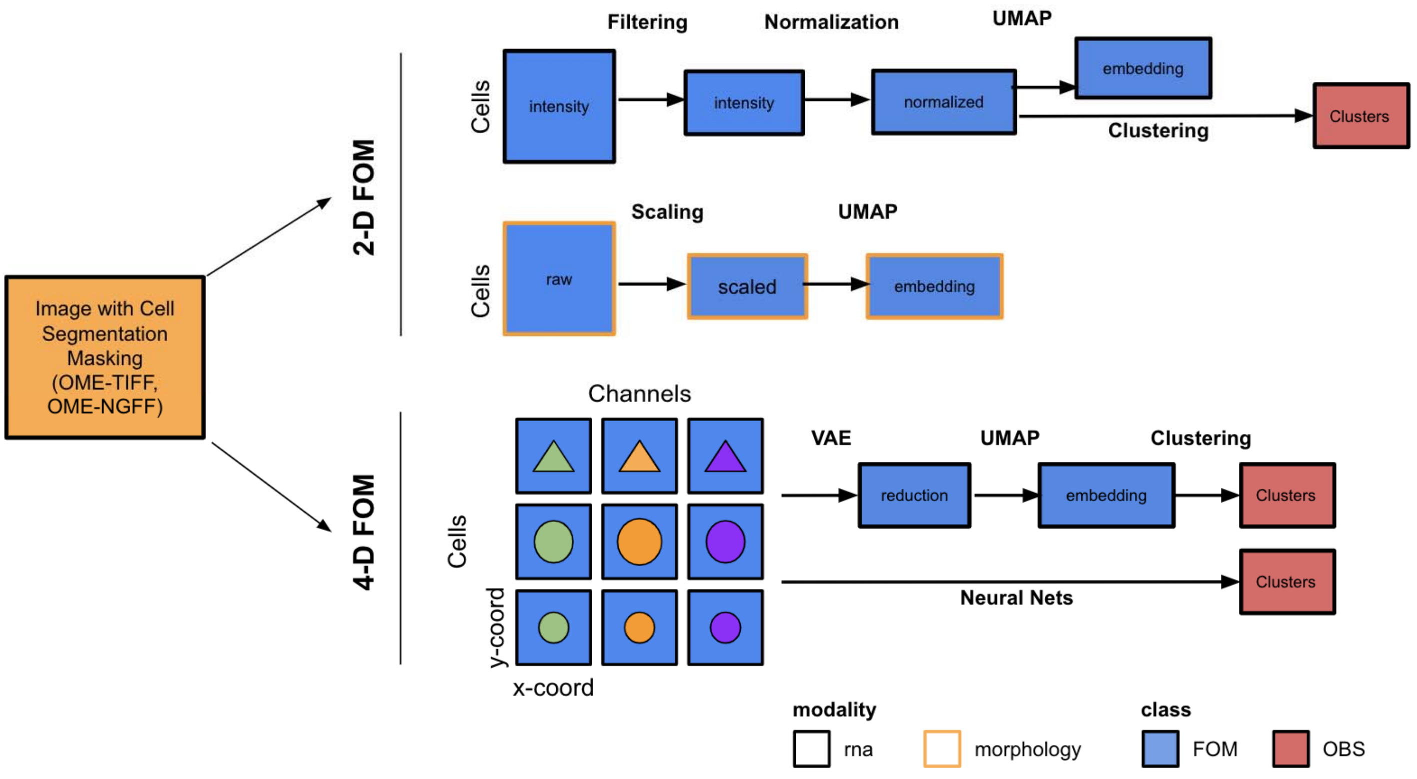

Supplementary Figure 4. Imaging-based analysis workflow that produces FOMs. Several imaging-based technologies can examine tissue slides to quantify molecular and cellular features in 2-D or 3-D space. Image analysis methods (e.g., segmentation) or manual curation by an expert can be used to identify Regions of Interest (ROIs) such as cell neighborhoods on each slide. Spatial technologies yield two flavors of FOMs. First, 2-dimensional FOMs can be derived that quantify the intensity levels of each marker in each cell or ROI. These raw intensity matrices can be augmented by additional morphological characteristics, such as cell size and shape, as well as spatial motifs within ROIs. The 2D FOMs can be filtered, normalized and used in clustering and embedding workflows in a similar fashion to single-cell sequencing FOMs. Second, a 4-D FOM can be generated that stores the pixel-level intensities along the x- and y-coordinates in each cell or ROI for each channel/marker. This type of FOM can be analyzed with algorithms that work directly on pixel-level data, such as variational autoencoders, to produce a reduced dimensional matrix which can be used in clustering and embedding workflows. Feature annotation and ID classes are not shown.
